# Pregnancy feasibility in women with mild pulmonary arterial hypertension: a systematic review and meta-analysis

**DOI:** 10.1186/s12884-023-05752-w

**Published:** 2023-06-08

**Authors:** Ruilin Ma, Hui Gao, Jianjian Cui, Haoran Shi, Zejun Yang, Zhishan Jin, Xiaoxia Liu, Di Wu, Weifang Liu, Yanfang Zheng, Yin Zhao

**Affiliations:** 1 Union Hospital, Tongji Medical College, Huazhong University of Science and Technology, Wuhan, China; 2 The First Affiliated Hospital of Zhengzhou University, Zhengzhou, China; 3 Shenzhen Huazhong University of Science and Technology Research Institute, Shenzhen, China

**Keywords:** Pregnancy, Pulmonary arterial hypertension, Systematic review and meta-analysis, Pregnancy, High risk pregnancy

## Abstract

**Background:**

The aim of this study was to evaluate the pregnancy feasibility of women with mild pulmonary hypertension according to pregnancy outcomes.

**Methods:**

This systematic review and meta-analysis compared the differences in maternal and fetal outcomes between mild and moderate-to-severe pulmonary hypertension. Relevant English and Chinese literature were searched in the PubMed, Embase, Cochrane Central Register of Controlled Trials (COCHRANE), CNKI, WanFang Data, and VIP databases between January 1st, 1990 and April 18th, 2023, and the references of the included articles and relevant systematic reviews were reviewed to determine whether studies were missed. The inclusion criteria were randomized controlled and observational studies (including case-control studies and cohort studies) examining maternal and fetal pregnancy outcomes with pulmonary hypertension. Conference abstracts, case reports, case series reports, non-comparative studies, and review articles were excluded.

**Results:**

This meta-analysis included 32 studies. In this study, maternal and fetal outcomes were better in the mild pulmonary hypertension group than in the moderate-to-severe group. Regarding maternal mortality, the mild group was much lower than the moderate to severe group. We found a significant decrease in maternal mortality in the mild group after 2010. However, no significant difference in maternal mortality before and after 2010 was observed in the moderate to severe group. Cardiac complications, ICU admission, neonatal preterm birth, small for gestational age infants, low birth weight infants, neonatal asphyxia, and neonatal mortality were significantly lower in the mild pulmonary hypertension group than in the moderate to severe pulmonary hypertension group. The cesarean section rates of the two groups were similar. However, the vaginal delivery rate in the mild pulmonary hypertension group was significantly higher than that in the moderate to severe pulmonary hypertension group.

**Conclusions:**

This meta-analysis confirmed that pregnancies with mild pulmonary hypertension had significantly better maternal and fetal outcomes than those with moderate to severe pulmonary hypertension. For patients with mild pulmonary hypertension and good cardiac function, continued pregnancy or even delivery should be considered under multidisciplinary monitoring. However, maternal and fetal complications with moderate to severe pulmonary hypertension significantly increase. Hence, it is essential to evaluate pregnancy risk and terminate it in time.

**Supplementary Information:**

The online version contains supplementary material available at 10.1186/s12884-023-05752-w.

## Background

Pulmonary arterial hypertension (PAH) is a syndrome characterized by elevated pulmonary artery pressure, which leads to obvious pulmonary artery remodeling and overload, eventually resulting in right ventricular hypertrophy, remodeling, and right heart failure [1]. According to the 2015 guidelines of the European Society of Cardiology (ESC) and the European Respiratory Society (ERS) [2, 3], the hemodynamics of pulmonary hypertension are defined as an elevated pulmonary artery pressure (mPAP) measured by right heart catheterization (RHC) to ≥ 25 mmHg at rest. In recent studies, it was further suggested that the mean pulmonary artery pressure should be defined as pulmonary artery hypertension if it exceeds 20 mmHg [1, 4, 5] .2022 ESC/ERS guideline [6] updated the hemodynamic definition of pulmonary hypertension and formally defined 20mmHg as the boundary of its diagnostic criteria. Pulmonary hypertension was divided into five groups according to different causes: pulmonary arterial hypertension, pulmonary hypertension caused by left heart disease, pulmonary hypertension caused by pulmonary disease hypoxia, pulmonary hypertension caused by pulmonary embolism, and other pulmonary hypertension of unknown causes or multiple factors [1, 3, 4].

Maternal hemodynamics is significantly altered during pregnancy. Increased blood volume and cardiac output, decreased systemic vascular resistance, increased oxygen consumption, and a hypercoagulable blood state exacerbates the risk of hypoxemia, pulmonary embolism and deep venous thrombosis [7]. For pregnant women with pulmonary hypertension, mothers and fetuses face greater challenges. According to the 2018 ESC of Cardiology guidelines for the Management of Cardiovascular Diseases during pregnancy, women with pulmonary hypertension should avoid pregnancy [8]. However, pulmonary hypertension may be diagnosed for the first time during pregnancy on rare occasions. With advancements in medical treatment, an increasing number of pregnant women with pulmonary hypertension are eager to continue their pregnancy and deliver a healthy newborn. Some studies have suggested that patients with good cardiac function and mild pulmonary hypertension can become pregnant under close multidisciplinary monitoring. However, it is currently debatable whether patients with mild pulmonary hypertension can continue their pregnancy or even have a vaginal delivery. Therefore, we used recent data to conduct this meta-analysis to compare the differences in maternal and fetal outcomes between pregnancies with mild PAH and moderate to severe PAH. To evaluate the pregnancy feasibility of women with mild pulmonary hypertension according to pregnancy outcomes.

## Methods

This Meta-analysis and report were conducted in accordance with the guidance of the Preferred Reporting Items for Systematic Reviews and Meta-Analyses (PRISMA). The search strategy, selection criteria, data extraction, quality evaluation, and statistical analysis were determined before the study and registered on PROSPERO on November 17, 2021, with registration number CRD42021284366.

### Article retrieval strategy and retrieval standard

The inclusion criteria will follow the principles of PECOS(Patient, Exposure, Comparison, Exposure, Outcome and Study designs). P is the pregnant woman. E is the patients diagnosed with mild pulmonary hypertension. C is moderate to severe pulmonary hypertension is maternal outcome: maternal mortality: vaginal delivery rate, cesarean section rate, ICU admission, and cardiac complications(occurrence of arrhythmia, infective endocarditis, infectious intima, cardiac failure, cardiogenic death, and pulmonary hypertension crisis) [9, 10] and fetal outcomes included birth weight, neonatal preterm birth, and small for gestational age infants, low birth weight infants, neonatal asphyxia, and neonatal mortality. All the outcomes are regarded as the primary outcomes. S is randomized controlled studies and observational studies including case-control and cohort studies.

The diagnostic criteria for pulmonary hypertension follows the criteria of the 2015 ESC/ERS guideline: mean pulmonary artery pressure ≥ 25 mmHg, pulmonary artery wedge pressure < 15 mmHg, pulmonary artery resistance >3WU and (and/or) indirect estimation of pulmonary artery systolic pressure (PASP) ≥ 30 mmHg (1 mmHg = 0.133 kPa) based on tricuspid regurgitation pressure difference or intracardiac shunt velocity measured by echocardiography. All included studies used noninvasive echocardiography to estimate pulmonary artery pressure because pregnant women could not participate in the invasive right heart floating catheter. Pulmonary hypertension is usually divided into mild and moderate to severe PAH, with a cutoff of 50 mmHg [3].The distinction between moderate and severe pulmonary hypertension is controversial. Both 70mmHg and 80mmHg have been reported as criteria for distinguishing moderate to severe, so this study mainly focuses on the comparative analysis of mild and moderate to severe.

Between January 1st, 1990, and April 18th, 2023, relevant English and Chinese literature published in PubMed, Embase, Cochrane Central Register of Controlled Trials (COCHRANE), CNKI, Wanfang Data and VIP databases were searched using the keywords “pregnancy” and “pulmonary hypertension,” and “maternal and fetal outcomes,” and the references of included articles and relevant systematic reviews were reviewed to determine whether any studies were missed. For the specific form, see Supplemental Materials.

### Research selection and data extraction

Two researchers independently completed data extraction and literature quality evaluation. After deleting duplicate literature, the article was filtered by reviewing the title and abstract using the same evaluation criteria. The available information for all included studies was recorded, including basic information (year of publication, first author, country implementing the study, sample size, and grouping criteria) and main outcome data (sample size, incidence, average, and standard deviation). Any disagreements or uncertainties were resolved through discussion with the help of a third reviewer. For the quality evaluation of the included studies, RCT and observational studies were evaluated using the Cochrane Intervention System Evaluation Manual (version 5.1.0) and the revised NIH-QAT, respectively.

### Statistical analysis

Risk ratio (RR) and 95% confidence interval (CI) were used to evaluate dichotomous variables. For continuous variables, the weighted mean difference (WMD) or standardized mean difference (SMD) and 95% confidence intervals were used for evaluation. When there are differences in the measurement methods or units of the outcome included in the literature, SMD is selected. The incidence of each result was analyzed using a one-arm meta-analysis. Meta-analysis was carried out using the random effects model and the Mantel-Haenszel method. Statistical significance was set at *p* < 0.05. In addition to calculating the estimated differences between the study groups, the statistical heterogeneity between the studies was calculated, with I^2^ > 50% indicating significant heterogeneity. When I^2^ was > 50%, the random effect model was used to analyze the results, whereas when I^2^ was < 50%, the fixed effect model was used. When the number of articles was greater than 10, the funnel chart test and Egger test were used to detect the effect of small studies on publication bias.

All statistical analyses were performed using Review Manager 5.4, STATA 16, and R Studio.

## Results

**Fig. 1:**
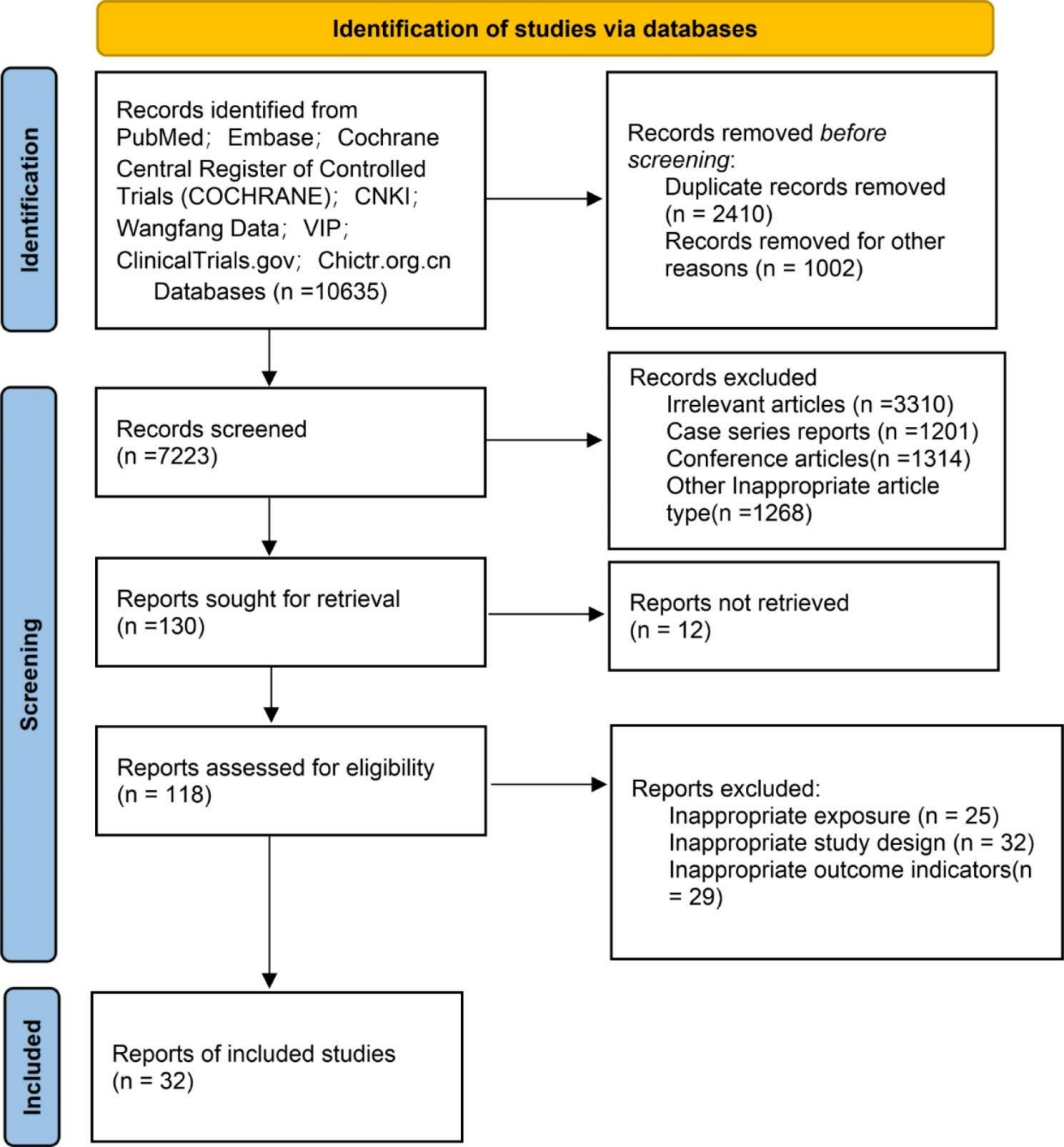
Flowchart of studies that met the inclusion criteria of meta-analysis

According to the retrieval strategy mentioned above, 10,635 articles were found on PubMed (n = 2,695), Cochrane (n = 609), Embase (n = 5452), CNKI(n = 757),Wanfang Data(n = 604),VIP(n = 518) and other websites. After deletion and repetition, 7223 records were retained. In addition,7093 records were excluded for irrelevant articles (n = 3310),case series reports (n = 1201),conference articles(n = 1314) and other inappropriate article type(n = 1268) by looking at the title and summary. Of the remaining records, 98 records were deleted for various reasons, the original reports could not be retrieved(n = 12), inappropriate exposure (n = 25),inappropriate study design (n = 32) and inappropriate outcome indicators(n = 29).Finally,32 studies [9–40] with 2520 patients met the inclusion criteria (Fig. 1).

A total of 2520 pregnant women were enrolled in the study, mainly in the Chinese population. Most of the causes of pulmonary hypertension is congenital heart disease, and a few of them are idiopathic pulmonary hypertension. The proportion of primary parturient is relatively high, and the age distribution is between 21 and 39 years old. The characteristics and demographic data included in the study are shown in Table 1 and Supplementary Tables 1, respectively (Supplementary Material).

**Table 1:** Basic characteristics of the studies included in the comparison of mild and moderate to severe pulmonary hypertension

author	year	Number of patients	population	study type	Age(mean, SD)	Nulli/multiparous(n)	Other medical problems(n)	NIH-QAT
CHEN Lin, et al. [25]	2014	62	Chinese	A retrospective cohort study	28,3	NR	NR	Fair
CHEN Yan, et al. [23]	2019	75	Chinese	A retrospective cohort study	Mild PAH = 29.89,5.66;Moderate PAH = 27.86, 5.01; Severe PAH = 28.33,5.52	NR	NR	Good
CHEN Yihong, et al. [11]	2012	37	Chinese	A retrospective cohort study	26.8,8.6	34/3	NR	Good
FAN Shu, et al. [31]	2020	47	Chinese	A retrospective cohort study	Mild PAH = 28.72,5.71;Moderate PAH = 31.62, 8.10; Severe PAH = 22.75,3.32	36/16	NR	Fair
GOOG Yunxia, et al. [12]	2009	53	Chinese	A retrospective cohort study	27,4	/12	NR	Fair
HAN Cha, et al. [13]	2013	118	Chinese	A retrospective cohort study	29,5	82/36	NR	Good
HAO Jinhong, et al. [14]	2013	36	Chinese	A retrospective cohort study	26.7,4.54	NR	NR	Fair
Katsuragi Shinji, et al. [28]	2012	24	Japanese	A cohort study	Mild PAH = 29.5,3.5;Severe PAH = 30.1,4.0	Mild PAH = 8/6;Severe PAH = 15/13	Hypertension: Mild PAH = 2;Severe PAH = 3; DM: Mild PAH = 1, Severe PAH = 3	Fair
Lai Weisi, et al. [26]	2021	88	Chinese	A retrospective cohort study	Moderate PAH = 28.6,6.3;Severe PAH = 28.1,5.7	NR	NR	Fair
LI Bin, et al. [15]	2013	102	Chinese	A retrospective cohort study	27,5	NR	NR	Fair
LI Ning, et al. [37]	2017	85	Chinese	A retrospective cohort study	32,7	NR	NR	Fair
LIN Feng, et al. [16]	2009	91	Chinese	A retrospective cohort study	25,3.2	73/18	NR	Fair
LIN Jian-hua, et al. [35]	2006	61	Chinese	A retrospective cohort study	NR	NR	NR	Fair
LIN Li, et al. [17]	2013	85	Chinese	A retrospective cohort study	26.2,8.5	NR	NR	Fair
LIN Xiao, et al. [32]	2020	151	Chinese	A retrospective cohort study	29.8,4.5	NR	NR	Fair
Luo Jun et al. [27]	2020	79	Chinese	A cohort study	Mild PH = 26.6,5.7;Severe PH = 26.0,4.9	Mild PH = 29/7;Severe PH = 33/10	NR	Good
Subbaiah Murali, et al. [9]	2013	30	Indian	A cohort study	Mild PAH = 28.64, 4.09;Severe PAH = 28.02, 3.9	Mild PAH = 10/6;Severe PAH = 8/6	Hypertension: Mild PAH = 1;Severe PAH = 0; Hypothyroidism: Mild PAH = 1; Severe PAH = 3; DM: Mild PAH = 2, Severe PAH = 1	Fair
WANG Wenjing, et al. [29]	2015	79	Chinese	A retrospective cohort study	26.3, 4.58	NR	NR	Fair
WEI Ming-zhu,et al. [36]	2009	44	Chinese	A retrospective cohort study	26, 3	33/11	NR	Fair
WU Yafeng, et al. [34]	2020	130	Chinese	A retrospective cohort study	28.36, 5.41	NR	NR	Fair
YANG Dong, et al. [18]	2012	44	Chinese	A retrospective cohort study	29, 5	44	NR	Fair
YE Jun, et al. [19]	2010	18	Chinese	A retrospective cohort study	NR	16/2	NR	Fair
YUE Jun, et al. [20]	2011	43	Chinese	A retrospective cohort study	28.3	41/2	NR	Fair
ZHANG Chengju, et al. [21]	2018	64	Chinese	A retrospective cohort study	26.73, 5.67	NR	NR	Good
ZHANG Xiao, et al. [22]	2021	52	Chinese	A retrospective cohort study	28.7, 4.1	47/5	NR	Fair
ZHAO Chuchu, et al. [30]	2020	74	Chinese	A retrospective cohort study	25.7,4.66	NR	NR	Fair
ZHOU Xiaorui, et al. [10]	2013	149	Chinese	A retrospective cohort study	29.3, 5.7	NR	NR	Good
Zhou Qian et al. [33]	2021	60	Chinese	A cohort study	Mild PH = 31.1, 2.3;Moderate PH = 29.3, 1.7; Severe PH = 26.2, 2.1	NR	NR	Good
Zhu Cai-Xia et al. [24]	2018	79	Chinese	A cohort study	Mild PH = 30.9,5.4;Moderate PH = 30.7, 4.62; Severe PH = 26.8, 2.75	Mild PH = 35/22;Moderate PH = 8/2; Severe PH = 7/4	Pre-eclampsia: Mild PH = 7;Moderate PH = 1;Severe PH = 1; GDM: Mild PH = 11; Moderate PH = 1;Severe PH = 1; PROM: Mild PH = 5; Moderate PH = 2;Severe PH = 0; Placenta previa: Mild PH = 4; Moderate PH = 0;Severe PH = 0; Previous cesarean: Mild PH = 7; Moderate PH = 0;Severe PH = 1	Good
Budhram S, et al. [38]	2022	185	South African	A retrospective study	Mild PH = 26(22–23),Moderate PH = 30(23–33),Severe PH = 27(26–33)	NR	HIV status: Mild PAH = 34;Moderate PAH = 28;Severe PAH = 8	Good
Su J Y, et al. [39]	2022	94	Chinese	A retrospective study	32.71,5.95	46/48	NR	Good
Zhang Lin, et al. [40]	2022	181	Chinese	A retrospective study	Mild PH = 29(33–37),Moderate PH = 26(31–37),Severe PH = 24(30–34)	Mild PH = 32/101,Moderate PH = 11/31,Severe PH = 24/49	Hypertension: Mild PH = 44; Moderate PH = 5; Severe PH = 7; Pre-eclampsia/eclampsia: Mild PH = 40; Moderate PH = 5; Severe PH = 4; HELLP syndrome :Mild PH = 1; Moderate PH = 0; Severe PH = 0; Diabetes mellitus: Mild PH = 10; Moderate PH = 1; Severe PH = 3; Autoimmune disease: Mild PH = 8; Moderate PH = 0; Severe PH = 3; Liver damage: Mild PH = 1; Moderate PH = 2; Severe PH = 2; Kindney injury: Mild PH = 5; Moderate PH = 1; Severe PH = 3; Others: Mild PH = 10; Moderate PH = 2; Severe PH = 3	Good

According to the NIH-QAT evaluation,11 articles were Good,21 articles were Fair. The main reason for the decline of evidence quality was that there was no mention of whether to measure the exposure for each person more than once and whether to control the confounding factors in the statistical analysis. In addition, some studies lack tools or methods to measure outcomes﻿ accurately and reliably. See Supplementary Table 2 (Supplementary Material) for details.

### Maternal outcomes

Maternal outcomes included maternal mortality, the incidence of cardiac complications, and ICU admission (Table 2).

**Table 2:** Summary of results of a meta-analysis comparing maternal outcomes in the mild and moderate to severe groups

Outcomes	No. Studies	No. patients	Heterogeneity(I^2^, %)	Model	Results
Maternal mortality	15	1204	0	FE	RR:0.19, 95%CI:0.10–0.35, *p*<0.00001
Cardiac complications	4	454	16	FE	RR:0.42, 95%CI:0.30–0.60, *p*<0.00001
ICU admission	4	269	0	FE	RR:0.37, 95%CI:0.26–0.53, *p* <0.00001

#### Maternal mortality

Overall, 15 studies [11–22, 38–40] were conducted, with 1,204 patients enrolled. Single-arm meta-analysis showed that the maternal mortality rates in the mild and moderate to severe groups were 0.15% (95%CI:0.00–0.93%) and 9% (95%CI:7–12%), respectively (Supplementary Fig. 1A and B in the Supplementary Material). Furthermore, mate analysis showed that the maternal mortality rate in the mild group was significantly lower than that in the moderate to severe group, which was only approximately one-fifth of that in the moderate to severe group (RR:0.19, 95%CI:0.10–0.35, *p* < 0.00001). There was no heterogeneity in the study results (I^2^ = 0%, *p* = 0.79) (Supplementary Fig. 1C in the Supplementary Material). Meanwhile, we performed a subgroup analysis using 2010 as the cutoff year and found that the maternal mortality rate decreased after that year compared to before in the mild group (before 2010:2.13%, 95%CI:0.00–6.20%; after 2010:0.08%,95%CI:0.00–0.87%). However, it did not change much in the moderate to severe group (before 2010:9.45%,95%CI:3–15%; after 2010:9.28%,95%CI:6–11%) (Supplementary Fig. 1D and E in the Supplementary Material).

#### Cardiac complications

Four studies [9, 10, 39, 40] were conducted, with 454 patients enrolled. Single-arm meta-analysis showed that the incidence of cardiac complications in pregnant women in the mild group and moderate to severe groups was 13% (95%CI:0–28%) and 41% (95%CI:17–64%), respectively (Supplementary Fig. 2A and B in the Supplementary Material). The incidence of cardiac complications in the mild group was significantly lower than that in the moderate to severe group, which was only approximately two-fifths of that in the moderate to severe group (RR:0.42, 95%CI:0.30–0.60, *p* < 0.00001). These results showed a small heterogeneity (I^2^ = 16%, *p* = 0.31>0.05) (Supplementary Fig. 2C in the Supplementary Material).

#### ICU admission

Four studies [21–24] were conducted, with 269 patients enrolled. Single-arm meta-analysis showed that the incidence of ICU admission in the mild group and the moderate to severe groups was 18% (95%CI: 2–34%) and 55% (95%CI: 2–9%), respectively (Supplementary Fig. 3A and B in the Supplementary Material). A meta-analysis showed that the incidence of ICU admission was much lower than that of the moderate to the severe group, which was only approximately one-third of that of the moderate to severe group (RR:0.37, 95%CI: 0.26–0.53, *p* < 0.00001). There was no heterogeneity in the research results (I^2^ = 0%, p = 0.73) (Supplementary Fig. 3C in the Supplementary Material).

### Fetal outcomes

Maternal outcomes included birth weight, week of delivery, neonatal preterm birth, and small for gestational age infants, low birth weight infants, neonatal asphyxia, and neonatal mortality (Table 3).

**Table 3:** Summary of results of a meta-analysis comparing neonatal outcomes in the mild and moderate to severe groups

Outcomes	No. Studies	No. patients	Heterogeneity(I^2^, %)	Model	Results
Birth weight	14	826	61	RE	SMD:0.99, 95%CI:0.84–1.14, *p* <0.00001
Weeks of delivery	12	806	50	FE	SMD:0.88, 95%CI:0.72–1.03, *p* <0.00001
Neonatal preterm birth	24	1929	57	RE	RR:0.50, 95%CI:0.40–0.63, *p* <0.00001
Small for gestational age infants	8	556	16	FE	RR:0.25, 95%CI:0.17–0.38, *p* <0.00001
Low birth weight infants	5	553	53	RE	RR:0.63, 95%CI:0.39–1.06, *p* = 0.009<0.05
Neonatal asphyxia	19	1379	37	FE	RR:0.37, 95%CI:0.28–0.50, *p* <0.00001
Neonatal mortality	16	1306	0	FE	RR:0.29, 95%CI:0.16–0.54, *p* <0.0001

#### Birth weight

Overall, 14 studies [9, 14, 15, 17, 19, 21, 23, 25–30, 39] were conducted, with 826 patients enrolled. The results of the meta-analysis showed that the birth weight of newborns in the mild group was similar with that in the moderate to severe group (SMD: 0.99, 95%CI: 0.84–1.14, *p* < 0.00001). However, there was great heterogeneity in the research conclusion (I^2^ = 61%, *p* = 0.0010) (Supplementary Fig. 4A in the Supplementary Material).

#### Week of delivery

Overall, 12 studies [9, 17, 21–24, 26–28, 31–33] were conducted, with 806 patients enrolled. The gestational weeks of the mild group were larger than those of the moderate to severe group (SMD: 0.88, 95%CI: 0.72–1.03, *p* < 0.00001). However, this result was heterogeneous (I^2^ = 50%, *p* = 0.02) (Supplementary Fig. 4B in the Supplementary Material).

#### Neonatal preterm birth

Overall, 24 studies [10–20, 22–25, 27, 29, 30, 34, 35, 38–40] were conducted, with 1,929 patients enrolled. The incidence of neonatal preterm birth in the mild group and the moderate to severe groups was 20% (95%CI: 16–26%) and 43% (95%CI: 37–49%), respectively (Supplementary Fig. 5A and B in the Supplementary Material). The meta-analysis results showed that the incidence of neonatal preterm birth in the mild group was significantly lower than that in the moderate to severe group, which was only half of that in the moderate to severe group (RR: 0.50, 95%CI: 0.40–0.63, *p* < 0.00001). The study result was heterogeneous (I^2^ = 57%, *p* = 0.0003) (Supplementary Fig. 5C in the Supplementary Material).

#### Small for gestational-age infants

Eight studies [9, 12, 13, 16, 18, 24, 27, 28] were conducted, with 556 patients enrolled. A single-arm meta-analysis showed that the incidence of small for gestational age infants in the mild group and the moderate to the severe group was 6% (95%CI: 3–9%)and 40% (95%CI: 29–51%), respectively (Supplementary Fig. 6A and B in the Supplementary Material). The incidence of small for gestational age infants in the mild group was significantly lower than that in the moderate to the severe group, which was only a quarter of that in the moderate to severe group (RR: 0.25, 95%CI: 0.17–0.38, *p* < 0.00001), and the heterogeneity of the results was small (I^2^ = 16%, *p* = 0.31) (Supplementary Fig. 6C in the Supplementary Material).

#### Low birth weight infants

Five studies [11, 15, 24, 38, 40] were conducted, with 553 patients enrolled. Single-arm meta-analysis showed that the incidence of low birth weight infants in the mild group and the moderate to the severe group was 18%(95%CI: 9–28%) and 34% (95%CI: 22–46%), respectively (Supplementary Fig. 7A and B in the Supplementary Material). The incidence of low birth weight infants in the mild group was lower than that in the moderate to the severe group, which was approximately two-thirds of that in the moderate to severe group (RR:0.64, 95%CI:0.39–1.06, *p* = 0.009). However, there was some heterogeneity in the results (I^2^ = 53%, *p* = 0.08) (Supplementary Fig. 7C in the Supplementary Material).

#### Neonatal asphyxia

Overall, 19 studies [11–13, 15, 17, 19, 20, 24–27, 29, 30, 32, 34–36, 39] were conducted, with 1,379patients enrolled. Single-arm meta-analysis showed that the incidence of neonatal asphyxia in the mild group and the moderate to the severe group was 7% (95%CI:5–9%) and 17% (95%CI: 12–22%), respectively (Supplementary Fig. 8A and B in the Supplementary Material). The incidence of neonatal asphyxia the mild group was significantly lower than that of the moderate to severe group, which was approximately two-fifths of the moderate to severe group (RR: 0.37, 95%CI: 0.28–0.50, *p* < 0.00001). However, there was some heterogeneity in the results (I^2^ = 37%, *p* = 0.05) (Supplementary Fig. 8C in the Supplementary Material).

#### Neonatal mortality

Overall, 16 studies [11–13, 15–17, 19, 22, 24, 26, 27, 29, 30, 32, 39, 40] were conducted, with 1,306 patients enrolled. The neonatal mortality rates in the mild group and the moderate to severe groups were 1% (95%CI: 0–1%) and 6% (95%CI: 3–15%), respectively (Supplementary Fig. 9A and B in the Supplementary Material). The neonatal mortality rate in the mild group was significantly lower than that in the moderate to severe group (RR: 0.29, 95%CI: 0.16–0.54, *p* < 0.0001), and the results were not heterogeneous (I^2^ = 0%, *p* = 0.92) (Supplementary Fig. 9C in the Supplementary Material).

### Mode of delivery

The mode of delivery mainly includes vaginal and cesarean sections (Table 4).

**Table 4:** Summary of results of a meta-analysis comparing the mode of delivery in the mild and moderate to severe groups

Outcomes	No. Studies	No. patients	Heterogeneity(I^2^, %)	Model	Results
Vaginal delivery	20	1612	34	FE	RR:2.55, 95%CI:1.89–3.43, *p* <0.00001
Cesarean section.	22	1811	77	RE	RR:0.99, 95%CI:0.90–1.08, *p* = 0.81>0.05

#### Vaginal delivery

Overall, 20 studies [9, 10, 12, 13, 15–18, 23, 26, 27, 31, 32, 34, 36–39] were conducted, with 1,612 patients enrolled. The vaginal delivery rates in the mild group and the moderate to severe groups were 16% (95%CI: 10–24%) and 7% (95%CI: 5–9%), respectively (Supplementary Fig. 10A and B in the Supplementary Material). Meta-analysis showed that the vaginal delivery rate in the mild group was significantly higher than that in the moderate to severe group, approximately two and a half times that in the moderate to severe group (RR: 2.55, 95%CI: 1.89–3.43, *p* < 0.00001), and the heterogeneity of the results were low (I^2^ = 34%, p = 0.07) (Supplementary Fig. 10C in the Supplementary Material).

#### Cesarean section

Data from 22 studies [9, 12–19, 22–24, 26–28, 31, 32, 34, 36–40] with 1811 patients were included in the study. A single-arm meta-analysis showed that the cesarean section rates in the mild group and the moderate to severe groups were 80% (95%CI: 71–86%) and 79% (95%CI: 72–86%), respectively (Supplementary Fig. 11A and B in the Supplementary Material). The meta-analysis showed no statistical significance in the cesarean section rate in the mild and moderate to severe groups (RR: 0.99, 95%CI: 0.90–1.08, *p* = 0.81>0.05). There was great heterogeneity in the research results (I^2^ = 77%, *p* < 0.00001) (Supplementary Fig. 11C in the Supplementary Material).

### Other outcomes

This study also investigated other outcomes: maternal New York Heart Association(NYHA) III–IV during pregnancy and the incidence of general anesthesia for surgery (Table 5).

**Table 5:** Summary of results of a meta-analysis comparing other outcomes in the mild and moderate to severe groups

Outcomes	No. Studies	No. patients	Heterogeneity(I^2^, %)	Model	Results
Maternal NYHA III-IV during pregnancy	23	1876	28	FE	RR:0.26, 95%CI:0.21–0.31, *p* <0.00001
General anesthesia	7	569	61	RE	RR:0.72, 95%CI:0.49–1.06, *p* = 0.10>0.05

#### Maternal NYHA III–IV during pregnancy

Overall, 23 studies [13–16, 18–20, 23–27, 29–37, 39, 40] were conducted, and 1,876 patients were included in the study. The ratio of maternal NYHA III–IV during pregnancy in the mild group and the moderate to the severe group was 12% (95%CI: 8–16%) and 55% (95%CI: 49–61%), respectively (Supplementary Fig. 12A and B in the Supplementary Material). Maternal NYHA III–IV ratio during pregnancy in the mild group was significantly lower than that in the moderate to severe group, which was only one-third of that in the moderate to severe group (RR 0.26; 95%CI, 0.21–0.31, *p* < 0.00001). There was a small heterogeneity in the research results (I^2^ = 28%, *p* = 0.11) (Supplementary Fig. 12C in the Supplementary Material).

#### General anesthesia

Seven studies [22–24, 26–28, 40] were conducted, and 569 patients were included in the study. The use rate of general anesthesia during cesarean section in the mild group and the moderate to severe groups was 30% (95%CI: 5–55%) and 40% (95%CI:17–63%), respectively (Supplementary Fig. 13A and B in the Supplementary Material). There was no significant difference in the use rate of general anesthesia during cesarean section between the mild and the moderate-to-severe groups (RR: 0.72, 95%CI: 0.49–1.06, *p* = 0.10>0.05). However, there was some heterogeneity in the results (I^2^ = 61%, *p* = 0.02) (Supplementary Fig. 13C in the Appendix).

#### Publish deviation and sensitivity analysis

Funnel plots and Egger test results of all studies are shown in the appendix. Most of the studies had a funnel diagram symmetrical distribution. There might be potential publication bias between some studies, as shown by the Egger test. However, further pruning and supplementing this part of the study confirmed the robustness of the research results, indicating that the conclusions of this study are credible.

14 outcomes included in this Meta analysis are evaluated by grade. The results show that small for gestational age infants, neonatal asphyxia and neonatal mortality are moderate-quality evidence. Maternal mortality, cardiac complications, ICU admission and vaginal delivery are low-quality evidence. Birth weight, weeks of delivery, neonatal preterm birth, low birth weight infants, cesarean section, general anesthesia and maternal NYHA III-IV during pregnancy are very low-quality evidence. The main reasons for its quality damage are the possible existence of publication bias and great heterogeneity. And all included studies were retrospective and some of the study designs had limitations. The results are shown in Table 6.

**Table 6:** The quality of evidence of the studied outcomes

Summary of findings:						
Mild PAH compared to Moderate to severe PAH for Pregnant women						
**Patient or population**: Pregnant women **Setting**: **Exposure**: Mild PAH **Comparison**: Moderate to severe PAH						
Outcome № of participants (studies)	Relative effect (95% CI)	**Anticipated absolute effects (95% CI)**			Certainty	What happens
		Risk with Mild PAH	Risk with Moderate to severe PAH	**Difference**		
Maternal Death № of participants: 1204 (15 observational studies)	**RR 0.19** (0.10 to 0.35)	10.7%	**2.0%** (1.1 to 3.7)	**8.6% fewer** (9.6 fewer to 6.9 fewer)	⨁⨁◯◯ Low^a,b^	Maternal mortality was significantly lower in the Mild PAH
Cardiac complications № of participants: 454 (4 observational studies)	**RR 0.42** (0.30 to 0.60)	46.0%	**19.3%** (13.8 to 27.6)	**26.7% fewer** (32.2 fewer to 18.4 fewer)	⨁⨁◯◯ Low^a^	The incidence of cardiac complications was significantly lower in the Mild PAH
ICU admission № of participants: 269 (4 observational studies)	**RR 0.37** (0.26 to 0.53)	58.3%	**21.6%** (15.2 to 30.9)	**36.7% fewer** (43.1 fewer to 27.4 fewer)	⨁⨁◯◯ Low^a^	The incidence of ICU admission was significantly lower in the Mild PAH
Birth weight № of participants: 826 (14 observational studies)	-	-	-	SMD **0.99 higher** (0.84 higher to 1.14 higher)	⨁◯◯◯ Very low^a,b,c^	The birth weight of newborns was similar in the Mild PAH.
Week of delivery № of participants: 806 (12 observational studies)	-	-	-	SMD **0.88 higher** (0.72 higher to 1.03 higher)	⨁◯◯◯ Very low^a,b,c^	The birth weight of newborns was larger in the Mild PAH.
Premature № of participants: 1929 (24 observational studies)	**RR 0.50** (0.40 to 0.63)	43.6%	**21.8%** (17.4 to 27.5)	**21.8% fewer** (26.2 fewer to 16.1 fewer)	⨁◯◯◯ Very low^a,c^	The incidence of neonatal preterm birth was lower in the Mild PAH.
SGA № of participants: 556 (8 observational studies)	**RR 0.25** (0.17 to 0.38)	39.4%	**9.8%** (6.7 to 15)	**29.5% fewer** (32.7 fewer to 24.4 fewer)	⨁⨁⨁◯ Moderate	The incidence of small for gestational age infants was lower in the Mild PAH.
low birth weight № of participants: 553 (5 observational studies)	**RR 0.64** (0.39 to 1.06)	33.9%	**21.7%** (13.2 to 35.9)	**12.2% fewer** (20.7 fewer to 2 more)	⨁◯◯◯ Very low^c^	The incidence of low birth weight infants was lower in the Mild PAH.
Neonatal asphyxia № of participants: 1379 (19 observational studies)	**RR 0.37** (0.28 to 0.50)	19.5%	**7.2%** (5.5 to 9.8)	**12.3% fewer** (14 fewer to 9.8 fewer)	⨁⨁⨁◯ Moderate	The incidence of neonatal asphyxia was lower in the Mild PAH.
Neonatal death № of participants: 1306 (16 observational studies)	**RR 0.29** (0.16 to 0.54)	5.5%	**1.6%** (0.9 to 3)	**3.9% fewer** (4.7 fewer to 2.6 fewer)	⨁⨁⨁◯ Moderate	Neonatal mortality was lower in the Mild PAH.
Vaginal Delivery № of participants: 1612 (20 observational studies)	**RR 2.55** (1.89 to 3.43)	7.0%	**17.8%** (13.2 to 23.9)	**10.8% more** (6.2 more to 16.9 more)	⨁⨁◯◯ Low^b^	The vaginal delivery rate was higher in Mild PAH.
Cesarean section № of participants: 1811 (22 observational studies)	**RR 0.99** (0.90 to 1.08)	76.9%	**76.1%** (69.2 to 83)	**0.8% fewer** (7.7 fewer to 6.2 more)	⨁◯◯◯ Very low^b,d^	The cesarean section rate was similar in Mild PAH.
NYHA class III-IV № of participants: 1876 (23 observational studies)	**RR 0.26** (0.21 to 0.31)	54.0%	**14.0%** (11.3 to 16.7)	**40.0% fewer** (42.6 fewer to 37.3 fewer)	⨁◯◯◯ Very low^a,b,c^	Maternal NYHA III–IV ratio during pregnancy was lower in Mild PAH.
general anesthesia № of participants: 569 (7 observational studies)	**RR 0.72** (0.49 to 1.06)	36.0%	**25.9%** (17.6 to 38.1)	**10.1% fewer** (18.3 fewer to 2.2 more)	⨁◯◯◯ Very low^a^	The use rate of general anesthesia during cesarean section was lower in Mild PAH.

*The risk in the intervention group (and its 95% confidence interval) is based on the assumed risk in the comparison group and the **relative effect** of the intervention (and its 95% CI).

**CI**: confidence interval; **RR**: risk ratio; **SMD**: standardised mean difference

**GRADE Working Group grades of evidence**

**High certainty**: we are very confident that the true effect lies close to that of the estimate of the effect.

**Moderate certainty**: we are moderately confident in the effect estimate: the true effect is likely to be close to the estimate of the effect, but there is a possibility that it is substantially different.

**Low certainty**: our confidence in the effect estimate is limited: the true effect may be substantially different from the estimate of the effect.

**Very low certainty**: we have very little confidence in the effect estimate: the true effect is likely to be substantially different from the estimate of effect.

**Explanations**

a. There are certain limitations in design or implementation

b. There is a degree of publishing bias

c. Heterogeneity was large

d. Heterogeneity was very large

## Discussion

Pregnancy with pulmonary hypertension has always been a critical illness in obstetrics, as it is one of the diseases with the highest risk for maternal and neonatal death. According to research, the maternal mortality rate of pregnancy with pulmonary hypertension is as high as 6–25%, and the neonatal mortality rate is 8–36%.It is reported that without intervention, the maternal mortality rate is as high as 25-56% [41, 42], and the main cause of death is heart failure. Maternal Mortality due to Pulmonary Hypertension in the United States between 2001 and 2010 showed an upward trend yearly (5.5/100,000 in 2001 to 6.5/100,000 in 2010) [3, 9]. However, most studies have shown that the mortality rate of pregnancies complicated by pulmonary hypertension shows a downward trend worldwide [27, 43–45]. This meta-analysis showed that the overall mortality rate of pregnant women with mild pulmonary hypertension was approximately 0.15%, and subgroup analysis after 2010 showed that the maternal mortality rate after 2010 was significantly lower than that before 2010 (2.13% before 2010 and 0.08% after 2010). Compared with the global maternal mortality rate (0.22%) [46], the gap is small, which may indicate that with the improved global level of medical care, the use of cardiopulmonary replacement devices, and improved medical awareness, pregnant women with pulmonary hypertension can detect the disease earlier and implement timely interventions to reduce mortality. This meta-analysis study suggests that patients with mild pulmonary hypertension can still get pregnant. However, the maternal mortality in moderate to severe PH (9%) is much higher than global maternal mortality (0.22%) [46]. Women with severe PH is still considered unsafe for pregnancy. The results of this study suggest that the maternal mortality rate of moderate to severe PAH is lower than that of severe PAH patients reported in other literature (12.5-33%) [44, 47, 48]. The reason may be that the moderate to severe group includes not only severe but also some moderate PAH patients. Timely termination of pregnancy or cardiopulmonary bypass intervention, some moderate patients also showed a better pregnancy outcome. In addition, severe patients are often promptly identified and treated effectively due to the earlier onset of clinical symptoms. At the same time, with improved treatment technology around the world, more serious cases are eliminated before the disease progresses.

In addition to maternal mortality, pregnant women with pulmonary hypertension still face other cardiac risks, including deterioration of cardiac function and malignant arrhythmia. This may be related to changes in cardiovascular physiology during pregnancy. Increased blood volume and cardiac output are the most significant changes during pregnancy, which can be increased from 50% to 70% of those before pregnancy [49–51]. Due to the high pulmonary vascular resistance, patients with pulmonary hypertension cannot tolerate these changes, resulting in excessive right ventricular afterload. This eventually induces adverse cardiac events such as deterioration of cardiac function, pulmonary hypertension crisis, and heart failure [52, 53]. This meta-analysis showed that the incidence of cardiac complications in the moderate to severe group was three times that in the mild group. Furthermore, the proportion of grade III–IV NYHA in the mild group was lower than that in the moderate to severe group in this study. From this study, we discovered that the pregnancy risk of pregnant women with severe PAH is much higher than that in the mild group, indicating that the cardiac function and disease severity are closely related to the degree of pulmonary hypertension. The higher the pulmonary artery pressure, the worse the prognosis.

Pregnant women are affected by pulmonary hypertension, which adversely impacts newborns. Placental blood perfusion in the anoxic state affects the growth and development of the fetus due to the lack of blood oxygen. This may lead to fetal growth restriction, distress, and even death. Hence, the incidence of induced abortion, preterm delivery, and low birth weight infants has increased significantly [54]. In terms of birth weight, gestational age, premature delivery, the incidence of small for gestational age, low birth weight, neonatal asphyxia, and neonatal mortality, the moderate to severe pulmonary hypertension group performed significantly worse than the mild group. In this study, the neonatal mortality rate in the mild pulmonary hypertension group was approximately 0.12%, which is lower than the worldwide neonatal mortality rate (13.9 cases per 1000 occurrences, approximately 1.39%) [55] and much lower than the neonatal mortality rate in the moderate to severe group in this study (4%). Therefore, it is suggested that pregnant women with mild pulmonary hypertension still have a high chance of delivering healthy newborns. In contrast, women with moderate to severe pulmonary hypertension have a greater risk of their newborns developing health problems.

The mode of delivery for pregnant women with pulmonary hypertension remains controversial. It is difficult for pregnant women with pulmonary hypertension to withstand the change of cardiovascular pressure during vaginal delivery. In addition, accurate and good hemodynamic monitoring during delivery is also very important for such patients. Previously, cesarean section was a preferred method for pregnant women with pulmonary hypertension. Cesarean section enables such patients to pass the delivery period better and more safely. Therefore, the cesarean section rate of such patients is higher. However, current studies suggest that when pulmonary arterial pressure is well controlled and the second stage of labor is shortened, pulmonary arterial hypertension is not an absolute contraindication in vaginal delivery in pregnant women. Furthermore, according to the latest guidelines from the European Society of Cardiology, elective cesarean or vaginal delivery is less risky than intrapartum or emergency cesarean delivery [3, 56]. The findings of this meta-analysis revealed no statistical difference in the cesarean section rate between the mild and moderate to severe groups. In contrast, the vaginal delivery rate in the mild group was three times that of the moderate to severe group, suggesting that pregnant women with mild pulmonary hypertension chose and had a successful vaginal delivery.

The choice of intraoperative anesthesia is also worth studying. Previous studies have suggested that there may be a relationship between general anesthesia and maternal mortality. The mortality rate was four times higher in patients who received general anesthesia than in those who received regional anesthesia [57]. Tracheal intubation and positive pressure ventilation during general anesthesia may result in increased intrathoracic and pulmonary artery pressures, reducing venous return and causing changes in systemic hemodynamics and increased cardiac load, ultimately leading to irreversible cardiac failure [45, 46, 58]. In this meta-analysis, we found no statistical difference in the rate of general anesthesia use between the mild and moderate to severe groups. Many factors may influence the choice of anesthesia. For example, some pregnant women may choose general anesthesia because of contraindications to intraspinal anesthesia. However, combined spinal-epidural anesthesia may be the best choice for pregnant women with pulmonary hypertension [58]. Patients in the mild group may have milder clinical manifestations and a better prognosis, which may increase their tolerance to non-general anesthesia methods such as combined spinal and epidural anesthesia. Consideration should be given to using non-general anesthesia in this group of patients in clinical practice.

### Advantages and limitations

This study has several advantages. This meta-analysis evaluated the feasibility of pregnancy in mild pulmonary hypertension and is the latest review comparing mild pulmonary arterial hypertension and moderate to severe pulmonary arterial hypertension, focusing on the maternal and infant outcomes of pregnant patients with pulmonary arterial hypertension. Our study also had some limitations. First, the included studies were all observational, with limited methodological quality, and the results of the report were inadequate or selective. Second, we excluded case reports and case series reports of pulmonary hypertension complicated pregnancies, which may have a certain selection bias. Additionally, some of the results of this study were moderately to highly heterogeneous. Finally, there may have been some variabilities in the included studies after excluding case reports, which resulted in a predominantly Chinese population. The level of medical technology and the quality of pregnancy care vary from country to country around the world. There are more pregnant women with pulmonary hypertension in China. In addition, the literature studies in other areas are mainly based on case reports or series reports, which do not meet inclusion criteria. Therefore, the majority of the literature ultimately included is from the Chines population, which may have a certain impact on the generalizability of the results of the study. It is necessary to study more people in the future.

## Conclusions

This meta-analysis further analyzed the maternal and fetal outcomes of pregnancy with pulmonary hypertension in recent decades and compared pregnancy outcomes between mild and moderate to severe pulmonary hypertension. Simultaneously, this meta-analysis confirmed that the maternal and fetal outcomes of pregnant women with mild pulmonary hypertension were significantly better than those with moderate to severe pulmonary hypertension. Maternal and fetal outcomes have improved in many women with mild pulmonary hypertension, thanks to advances in cardiac supportive care, the use of vasodilators, and the management of multidisciplinary teams in recent decades.

Various guidelines do not encourage pregnancy in women with pulmonary hypertension. However, continuous control of pulmonary hypertension during pregnancy, multidisciplinary, individualized treatment, and planned early pregnancy termination seems to improve maternal and fetal outcomes further. This gives pregnant women with pulmonary hypertension new hope. Continued pregnancy or even delivery should be considered under multidisciplinary monitoring for patients with mild pulmonary hypertension and good cardiac function. However, maternal and fetal complications with moderate to severe pulmonary hypertension are significantly increased; hence, it is necessary to evaluate pregnancy risk and terminate it on time. The findings of this study provide a basis for pre-pregnancy counseling and risk assessment for obstetricians.

## Electronic supplementary material

Below is the link to the electronic supplementary material.

Supplementary Material 1

Supplementary Material 2

Supplementary Material 3

Supplementary Material 4

Supplementary Material 5

Supplementary Material 6

Supplementary Material 7

Supplementary Material 8

Supplementary Material 9

Supplementary Material 10

Supplementary Material 11

Supplementary Material 12

Supplementary Material 13

Supplementary Material 14

Supplementary Material 15

Supplementary Material 16

Supplementary Material 17

Supplementary Material 18

Supplementary Material 19

Supplementary Material 20

Supplementary Material 21

Supplementary Material 22

## Data Availability

All data can be obtained from manuscript.

## List of Abbreviations

PAH: Pulmonary arterial hypertension
COCHRANE: Cochrane Central Register of Controlled Trials
RR: Risk ratio
CI: Confidence interval
WMD: Weighted mean difference
SMD: Standardized mean difference
NYHA: New York Heart Association.
