# Supplementary material for: Pregnancy feasibility in women with mild pulmonary arterial hypertension: a systematic review and meta-analysis
Source: BMC Pregnancy Childbirth. 2023 Jun 8;23:427. doi: 10.1186/s12884-023-05752-w (PMC10249223; doi:10.1186/s12884-023-05752-w)
Supplement: Supplementary file 22 — Supplementary Material 22 [file 12884_2023_5752_MOESM22_ESM.docx]

**Supplemental Materials**

**Appendix S I**

**Search Strategy and Selection Criteria**

The search items included ("Pregnancy"[MeSH Terms] OR ("Pregnancy"[Title/Abstract] OR "pregnancies"[Title/Abstract] OR "gestation"[Title/Abstract] OR "pregnant women"[Title/Abstract])) AND ("hypertension, pulmonary"[MeSH Terms] OR ("hypertension pulmonary"[Title/Abstract] OR "pulmonary hypertension"[Title/Abstract] OR "pulmonary vasodilator"[Title/Abstract]) OR ("Pulmonary Arterial Hypertension"[MeSH Terms] OR ("Pulmonary Arterial Hypertension"[Title/Abstract] OR "arterial hypertension pulmonary"[Title/Abstract] OR "hypertension pulmonary arterial"[Title/Abstract])) OR ("Familial Primary Pulmonary Hypertension"[MeSH Terms] OR ("Familial Primary Pulmonary Hypertension"[Title/Abstract] OR "heritable pulmonary arterial hypertension"[Title/Abstract] OR (("hypertension, pulmonary"[MeSH Terms] OR ("Hypertension"[All Fields] AND "Pulmonary"[All Fields]) OR "pulmonary hypertension"[All Fields] OR ("Pulmonary"[All Fields] AND "Hypertension"[All Fields])) AND "primary 1"[Title/Abstract]) OR (("Familial Primary Pulmonary Hypertension"[MeSH Terms] OR ("familial"[All Fields] AND "Primary"[All Fields] AND "Pulmonary"[All Fields] AND "Hypertension"[All Fields]) OR "Familial Primary Pulmonary Hypertension"[All Fields] OR ("Pulmonary"[All Fields] AND "Hypertension"[All Fields] AND "Primary"[All Fields]) OR "pulmonary hypertension primary"[All Fields]) AND "Dexfenfluramine-Associated"[Title/Abstract]) OR (("Familial Primary Pulmonary Hypertension"[MeSH Terms] OR ("familial"[All Fields] AND "Primary"[All Fields] AND "Pulmonary"[All Fields] AND "Hypertension"[All Fields]) OR "Familial Primary Pulmonary Hypertension"[All Fields] OR ("Pulmonary"[All Fields] AND "Hypertension"[All Fields] AND "Primary"[All Fields]) OR "pulmonary hypertension primary"[All Fields]) AND "Fenfluramine-Associated"[Title/Abstract]) OR "idiopathic pulmonary arterial hypertension"[Title/Abstract] OR "primary pulmonary hypertension"[Title/Abstract] OR "hypertension primary pulmonary"[Title/Abstract] OR "pulmonary hypertension primary"[Title/Abstract] OR "idiopathic pulmonary hypertension"[Title/Abstract] OR "hypertension idiopathic pulmonary"[Title/Abstract] OR "pulmonary hypertension idiopathic"[Title/Abstract] OR (("Familial Primary Pulmonary Hypertension"[MeSH Terms] OR ("familial"[All Fields] AND "Primary"[All Fields] AND "Pulmonary"[All Fields] AND "Hypertension"[All Fields]) OR "Familial Primary Pulmonary Hypertension"[All Fields]) AND "hereditary hemorrhagic telangiectasia"[Title/Abstract]) OR (("Familial Primary Pulmonary Hypertension"[MeSH Terms] OR ("familial"[All Fields] AND "Primary"[All Fields] AND "Pulmonary"[All Fields] AND "Hypertension"[All Fields]) OR "Familial Primary Pulmonary Hypertension"[All Fields] OR "pph1"[All Fields]) AND "with hht"[Title/Abstract]))) OR ("Eisenmenger Complex"[MeSH Terms] OR ("complex eisenmenger"[Title/Abstract] OR "eisenmenger s complex"[Title/Abstract] OR "complex eisenmenger s"[Title/Abstract] OR "eisenmengers complex"[Title/Abstract] OR "eisenmenger s syndrome"[Title/Abstract] OR "eisenmengers syndrome"[Title/Abstract] OR "syndrome eisenmenger s"[Title/Abstract] OR "eisenmenger syndrome"[Title/Abstract] OR "syndrome eisenmenger"[Title/Abstract])) OR ("Cyanosis"[MeSH Terms] OR ("Cyanosis"[Title/Abstract] OR "Cyanoses"[Title/Abstract])) OR ("heart defects, congenital"[MeSH Terms] OR ("defect congenital heart"[Title/Abstract] OR "abnormality heart"[Title/Abstract] OR "heart abnormality"[Title/Abstract] OR "congenital heart defect"[Title/Abstract] OR "heart malformation of"[Title/Abstract] OR "malformation of heart"[Title/Abstract] OR (("Abnormalities"[MeSH Subheading] OR "Abnormalities"[All Fields] OR "malformations"[All Fields] OR "congenital abnormalities"[MeSH Terms] OR ("Congenital"[All Fields] AND "Abnormalities"[All Fields]) OR "congenital abnormalities"[All Fields] OR "Malformation"[All Fields] OR "malformational"[All Fields] OR "malformities"[All Fields] OR "malformed"[All Fields]) AND "Hearts"[Title/Abstract]) OR "defects congenital heart"[Title/Abstract] OR "heart abnormalities"[Title/Abstract] OR "heart defect congenital"[Title/Abstract] OR "congenital heart disease"[Title/Abstract] OR "congenital heart diseases"[Title/Abstract] OR "disease congenital heart"[Title/Abstract] OR "congenital heart defects"[Title/Abstract])) OR ("Tricuspid Valve Insufficiency"[MeSH Terms] OR ("Tricuspid Valve Insufficiency"[Title/Abstract] OR "insufficiency tricuspid valve"[Title/Abstract] OR "valve insufficiency tricuspid"[Title/Abstract] OR "tricuspid valve regurgitation"[Title/Abstract] OR "regurgitation tricuspid valve"[Title/Abstract] OR "valve regurgitation tricuspid"[Title/Abstract] OR "tricuspid valve incompetence"[Title/Abstract] OR "incompetence tricuspid valve"[Title/Abstract] OR "valve incompetence tricuspid"[Title/Abstract] OR "tricuspid incompetence"[Title/Abstract] OR "incompetence tricuspid"[Title/Abstract] OR "tricuspid regurgitation"[Title/Abstract] OR "regurgitation tricuspid"[Title/Abstract])) OR ("Heart Failure"[MeSH Terms] OR ("cardiac failure"[Title/Abstract] OR "cardiac failure"[Title/Abstract] OR "heart decompensation"[Title/Abstract] OR "decompensation heart"[Title/Abstract] OR "heart failure right sided"[Title/Abstract] OR "heart failure right sided"[Title/Abstract] OR "right sided heart failure"[Title/Abstract] OR "right sided heart failure"[Title/Abstract] OR "myocardial failure"[Title/Abstract] OR "congestive heart failure"[Title/Abstract] OR "heart failure congestive"[Title/Abstract] OR "heart failure left sided"[Title/Abstract] OR "heart failure left sided"[Title/Abstract] OR "left sided heart failure"[Title/Abstract] OR "left sided heart failure"[Title/Abstract]))) AND ("Pregnancy Outcome"[MeSH Terms] OR ("Pregnancy Outcome"[Title/Abstract] OR "pregnancy outcomes"[Title/Abstract] OR "outcome pregnancy"[Title/Abstract] OR "outcomes pregnancy"[Title/Abstract]) OR ("Obstetric Labor Complications"[MeSH Terms] OR ("Obstetric Labor Complications"[Title/Abstract] OR (("compliances"[All Fields] OR "complicate"[All Fields] OR "complicated"[All Fields] OR "complicates"[All Fields] OR "complicating"[All Fields] OR "Complication"[All Fields] OR "complication s"[All Fields] OR "Complications"[MeSH Subheading] OR "Complications"[All Fields]) AND "obstetric labor"[Title/Abstract]) OR (("compliances"[All Fields] OR "complicate"[All Fields] OR "complicated"[All Fields] OR "complicates"[All Fields] OR "complicating"[All Fields] OR "Complication"[All Fields] OR "complication s"[All Fields] OR "Complications"[MeSH Subheading] OR "Complications"[All Fields]) AND "obstetric labor"[Title/Abstract]) OR (("labor s"[All Fields] OR "labored"[All Fields] OR "laborer"[All Fields] OR "laborer s"[All Fields] OR "laborers"[All Fields] OR "laboring"[All Fields] OR "labors"[All Fields] OR "labour"[All Fields] OR "work"[MeSH Terms] OR "work"[All Fields] OR "Labor"[All Fields] OR "labor, obstetric"[MeSH Terms] OR ("Labor"[All Fields] AND "Obstetric"[All Fields]) OR "obstetric labor"[All Fields] OR "laboured"[All Fields] OR "labourer"[All Fields] OR "labourers"[All Fields] OR "labouring"[All Fields] OR "labours"[All Fields]) AND "complication obstetric"[Title/Abstract]) OR (("labor s"[All Fields] OR "labored"[All Fields] OR "laborer"[All Fields] OR "laborer s"[All Fields] OR "laborers"[All Fields] OR "laboring"[All Fields] OR "labors"[All Fields] OR "labour"[All Fields] OR "work"[MeSH Terms] OR "work"[All Fields] OR "Labor"[All Fields] OR "labor, obstetric"[MeSH Terms] OR ("Labor"[All Fields] AND "Obstetric"[All Fields]) OR "obstetric labor"[All Fields] OR "laboured"[All Fields] OR "labourer"[All Fields] OR "labourers"[All Fields] OR "labouring"[All Fields] OR "labours"[All Fields]) AND "complications obstetric"[Title/Abstract]) OR "obstetric labor complication"[Title/Abstract] OR "labor complications"[Title/Abstract] OR "complication labor"[Title/Abstract] OR "labor complication"[Title/Abstract] OR "complications labor"[Title/Abstract])).

**Appendix S II**

**Meta- analysis results**

**Supplementary Figure 1** **Meta-analysis of maternal mortality.** A, Single-arm meta-analysis of maternal mortality in the mild group. B, Single-arm meta-analysis of maternal mortality in the moderate to severe group. C, Forest plots of meta-analysis of maternal mortality. D, Subgroup analysis of maternal mortality in the mild group. E, Subgroup analysis of maternal mortality in the moderate-to severe group.

**Supplementary Figure 2 Meta-analysis of cardiac complications.** A, Single-arm meta-analysis of cardiac complications in the mild group. B, Single-arm meta-analysis of cardiac complications in the moderate to severe group. C, Forest plots of meta-analysis of cardiac complications.

**Supplementary Figure 3 Meta-analysis of ICU admission**. A, Single-arm meta-analysis of ICU admission in the mild group. B, Single-arm meta-analysis of ICU admission in the moderate to severe group. C, Forest plots of meta-analysis of ICU admission.

**Supplementary Figure 4 Forest plot from meta-analysis of birth weight and week of delivery.** A, Birth weight. B, Week of delivery.

**Supplementary Figure 5 Meta-analysis of neonatal preterm birth rate.** A, Single-arm meta-analysis of neonatal preterm birth rate in the mild group. B, Single-arm meta-analysis of neonatal preterm birth in the moderate rate to severe group. C, Forest plots of meta-analysis of neonatal preterm birth rate.

 **Supplementary Figure 6 Meta-analysis of small for gestational-age infants.** A, Single-arm meta-analysis of incidence of small for gestational-age infants in the mild group. B, Single-arm meta-analysis of incidence of small for gestational-age infants in the moderate to severe group. C, Forest plots of meta-analysis of incidence of small for gestational-age infants.

**Supplementary Figure 7 Meta-analysis of low birth weight infants.** A, Single-arm meta-analysis of incidence of low birth weight infants in the mild group. B, Single-arm meta-analysis of incidence of low birth weight infants in the moderate to severe group. C, Forest plots of meta-analysis of incidence of low birth weight infants.

**Supplementary Figure 8 Forest plot from meta-analysis of neonatal asphyxia.** A, Single-arm meta-analysis of incidence of neonatal asphyxia in the mild group. B, Single-arm meta-analysis of incidence of neonatal asphyxia in the moderate to severe group. C, Forest plots of meta-analysis of incidence of neonatal asphyxia.

**Supplementary Figure 9 Meta-analysis of neonatal mortality.** A, Single-arm meta-analysis of neonatal mortality in the mild group. B, Single-arm meta-analysis of neonatal mortality in the moderate to severe group. C, Forest plots of meta-analysis of neonatal mortality.

**Supplementary Figure 10 Meta-analysis of vaginal delivery.** A, Single-arm meta-analysis of vaginal delivery rate in the mild group.B, Single-arm meta-analysis of vaginal delivery rate in the moderate to severe group.C, Forest plots of meta-analysis of vaginal delivery rate.

**Supplementary Figure 11 Meta-analysis of cesarean section.** A, Single-arm meta-analysis of cesarean section rate in the mild group.B, Single-arm meta-analysis of cesarean section rate in the moderate to severe group.C, Forest plots of meta-analysis of cesarean section rate.

**Supplementary Figure 12 Meta-analysis of maternal NYHA class III-IV rate during pregnancy.** A, Single-arm meta-analysis of rate of maternal NYHA class III-IV during pregnancy in the mild group.B, Single-arm meta-analysis of rate of maternal NYHA class III-IV during pregnancy in the moderate to severe group.C, Forest plots of meta-analysis of rate of maternal NYHA class III-IV during pregnancy.

**Supplementary Figure 13 Meta-analysis of rate of using general anesthesia for cesarean section.** A, Single-arm meta-analysis of rate of using general anesthesia for cesarean section in the mild group.B, Single-arm meta-analysis of rate of using general anesthesia for cesarean section in the moderate to severe group.C, Forest plots of meta-analysis of rate of using general anesthesia for cesarean section.

**Publish biased results**

**Supplementary Figure 14 A-B Publication bias of results comparing the mild and moderate to severe group.** A, Funnel plot of maternal mortality. B, Funnel plot of the trim-and-fill method of maternal mortality (egger test result: *p*=0.0787 > 0.05, combined results were robust after seven pieces of literature supplemented by the trim-and-fill method). **C-E Publication bias of single-arm meta-analysis.** C, Funnel plot of maternal mortality in the mild group(egger test result: *p*=0.1833> 0.05).D, Funnel plot of maternal mortality in the moderate to severe group(egger test result: *p*=0.0430<0.05).E, Funnel plot of the trim-and-fill method of maternal mortality in the moderate to severe group(Combined results were robust after four pieces of literature supplemented by the trim-and-fill method).

**Supplementary Figure 15 Publication bias of the results comparing the mild and moderate to severe group.** A, Funnel plot of birth weight(egger test Results: *p*=0.0028＜0.05). B, Funnel plot of the trim-and-fill birth weight (Combined results were robust after zero piece of literature supplemented by the trim-and-fill method). C, Funnel plot of week of delivery(egger test Results: *p*=0.0136＜0.05). D, Funnel plot of the trim-and-fill method of week of delivery(Five literatures were supplemented by the trim-and-fill method, the results were still not reversed, and the results were stable).

**Supplementary Figure 16 Publication bias of the results comparing the mild and moderate to severe group.** A, Funnel plot of neonatal preterm birth (egger test result: *p*=0.2057>0.05). **B-D Publication bias of single-arm meta-analysis.** B, Funnel plot of neonatal preterm birth in the moderate to severe group(egger test result: *p*=0.8034>0.05).C, Funnel plot of neonatal preterm birth in the mild group(egger test result: *p*＜0.0001).D, The funnel plot of the trim-and-fill method of neonatal preterm birth in the mild group (Eleven literatures were supplemented by the trim-and-fill method, the results were still not reversed, and the results were stable).

**Supplementary Figure 17 Publication bias of the results comparing the mild and moderate to severe group.** A, Funnel plot of neonatal asphyxia (egger test result: *p*=0.5660>0.05). **B-D Publication bias of single-arm meta-analysis.** B, Funnel plot of neonatal asphyxia in the mild group(egger test result: *p*=0.0048＜0.05).C, The funnel plot of the trim-and-fill method of neonatal asphyxia in the mild group(Seven literatures were supplemented by the trim-and-fill method, the results were still not reversed, and the results were stable).D, Funnel plot of neonatal asphyxia in the moderate to severe group(egger test result: *p*=0.0002＜0.05).D, The funnel plot of the trim-and-fill method of neonatal asphyxia in the moderate to severe group (Eight literatures were supplemented by the trim-and-fill method, the results were still not reversed, and the results were stable).

**Supplementary Figure 18 Publication bias of the results comparing the mild and moderate to severe group.** A, Funnel plot of neonatal mortality (egger test result: *p*=0.8687>0.05). **B-C Publication bias of single-arm meta-analysis.** B, Funnel plot of neonatal mortality in the mild group(egger test result: *p*=0.3404>0.05).C, Funnel plot of neonatal mortality in the moderate to severe group(egger test result: *p*=0.1279>0.05).

**Supplementary Figure 19 Publication bias of the results comparing the mild and moderate to severe group.** A, Funnel plot of vaginal delivery(egger test result: *p*=0.0354＜0.05). B, The funnel plot of the trim-and-fill method of vaginal delivery(The results of five literatures supplemented by the trim-and-fill method were still not reversed and the results were stable). **C-F Publication bias of single-arm meta-analysis.** C, Funnel plot of vaginal delivery in the mild group(egger test result: *p*=0.0490＜0.05). D, The funnel plot of the trim-and-fill method of vaginal delivery in the mild group(Five literatures were supplemented by the trim-and-fill method, the results were still not reversed, and the results were stable).E, Funnel plot of vaginal delivery in the moderate to severe group(egger test result: *p*＜0.0001).F, The funnel plot of the trim-and-fill method of vaginal delivery in the moderate to severe group (Eight literatures were supplemented by the trim-and-fill method, the results were still not reversed, and the results were stable).

**Supplementary Figure 20 A-B Publication bias of the results comparing the mild and moderate to severe group.** A, Funnel plot of cesarean section (egger test result: *p*=0.0354＜0.05). B, The funnel plot of the trim-and-fill method of cesarean section (The results of two literatures supplemented by the trim-and-fill method were still not reversed and the results were stable). **C-E Publication bias of single-arm meta-analysis.** C, Funnel plot of cesarean section in the mild group(egger test result: *p*=0. 1647＞0.05). D, Funnel plot of cesarean section in the moderate to severe group(egger test result: *p*＜0.001).E, The funnel plot of the trim-and-fill method of cesarean section in the moderate to severe group (Eleven literatures were supplemented by the trim-and-fill method, the results were still not reversed, and the results were stable).

**Supplementary Figure 21 A-B Publication bias of the results comparing the mild and moderate to severe group.** A, Funnel plot of maternal NYHA class III-IV rate during the pregnancy (egger test result: *p*=0.0017＜0.05). B, The funnel plot of the trim-and-fill method of maternal NYHA class III-IV rate during the pregnancy (The results of eight literatures supplemented by the trim-and-fill method were still not reversed and the results were stable). **C-E Publication bias of single-arm meta-analysis.** C, Funnel plot of maternal NYHA class III-IV rate during the pregnancy in the mild group(egger test result: *p*＜0.0001). D, The funnel plot of the trim-and-fill method of maternal NYHA class III-IV rate during the pregnancy in the mild group (Eleven literatures were supplemented by the trim-and-fill method, the results were still not reversed, and the results were stable).E, Funnel plot of maternal NYHA class III-IV rate during the pregnancy in the moderate to severe group(egger test result: *p*=0.3077＞0.05).

Supplementary Table 1 Basic characteristics of the studies included in the comparison of mild and moderate to severe pulmonary hypertension

| author | year | category of PAH | comparisons | outcomes |
| --- | --- | --- | --- | --- |
| CHEN Lin，et al. | 2014 | CHD：Mild PAH 13，Moderate PAH 10，Severe PAH 5  Rheumatic heart disease：Mild PAH 2，Moderate PAH 6,Severe PAH 7 | Mid PAH：sPAP 30~49 mmHg  Moderate PAH：sPAP 50~79 mmHg  Severe PAH：sPAP ≥80 mmHg | Class I-II、Class II-IV、Term of delivery；Premature、Neonatal asphyxia、Birth weight、Induced abortion |
| CHEN Yan，et al. | 2019 | Mild PAH:CHD 6,Rheumatic heart disease 2，HDCP 13,Connective tissue disease 2，IPAH 8，Others 5  Moderate PAH：CHD 5，HDCP 7，IPAH 3，Others 6  Severe PAH：CHD 8,Rheumatic heart disease 2，HDCP 5,Connective tissue disease 1，Others 2 | Mid PAH：SPAP 30~49mmHg  Moderate PAH：SPAP 50~69 mmHg  Severe PAH：SPAP ≥70 mmHg | Class I-II、Class II-IV、Mode of delivery、premature、Induced abortion、term of delivery、ICU admission、General anesthesia、Week of delivery、Birth weight、Neonatal asphyxia、NICU admission |
| CHEN Yihong，et al. | 2012 | Mild PAH=CHD(8)、Rheumatic heart disease(8)、Perinatal cardiomyopathy(2)、Hypertrophic cardiomyopathy(1)  Moderate PAH=CHD(1)、Rheumatic heart disease(7)、Perinatal cardiomyopathy(2)、  Severe PAH=CHD(2)、Rheumatic heart disease(3)、Perinatal cardiomyopathy(1)、IPAH(1)、Others(1) | Mild PAH：30-＜ 50 mmHg  Moderate PH：50-＜80 mmHg  Severe PH ： ≥80 mmHg  . | Class I-II、Class II-IV、Iuduced labour、Neonatal asphyxia、Neonatal Death、Premature、Maternal Death、Low Birth Weight、Term delivery |
| FAN Shu，et al. | 2020 | NR | Mild PAH ：PASP 30-49mmHg  Moderate PAH：PASP 50-79mmHg  Severe PAH：PASP ≥80mmHg | Week of delivery、Mode of delivery、Class I-II、Class II-IV、Mode of delivery、General anesthesia |
| GOOG Yunxia, et al. | 2009 | Rheumatic heart disease：12  CHD：24  PIAH：5  Peripartum cardiomyopathy：8  Hypertension heart disease：4 | slight PAH：PABP ≥30 and <50 mmHg  moderate PAH：PABP ≥50 and <80 mmHg  severe PAH： PABP ≥80 mmHg | Mode of delivery、SGA、Premature、Neonatal asphyxia、Maternal Death、Neonatal death、Term Delivery |
| HAN Cha，et al. | 2013 | Mild PAH=CHD(40)、Rheumatic heart disease(11)、Perinatal cardiomyopathy(1)、Preeclampsia（5）、Connective tissue disease(2)  Moderate PAH=CHD(20)、Rheumatic heart disease(7)、Perinatal cardiomyopathy(2)、Preeclampsia（7）、Connective tissue disease(1)、IPAH(1)  Severe PAH=CHD(8)、Rheumatic heart disease(4)、Perinatal cardiomyopathy(2)、Preeclampsia（4）、Connective tissue disease(2)、IPAH(1) | Mild PAH：30-49 mmHg  Moderate PH：50-79 mmHg  Severe PH ： ≥80 mmHg  . | Class I-II、Class II-IV、Term delivery、Premature、SGA、Mode of delivery、Induced labour、Maternal Death、Neonatal asphyxia、Neonatal Death |
| HAO Jinhong，et al. | 2013 | ASD | Mild PAH：36-49 mmHg  Moderate PH：50-69 mmHg  Severe PH ： ≥70 mmHg  . | Class I-II、Class II-IV、Term delivery、premature、Induced labour、Birth weight、Maternal Death、saturation of blood oxygen、partial pressure of blood oxygen |
| Katsuragi, Shinji et al. | 2012 | Mild PAH= IPAH(2),CHD:ASD(4),VSD(3),PDA(2),ECD(1),  Collagon disease（2）  Severe PAH=IPAH(5),CHD：ASD(2),VSD(3),PDA(2),  Eisenmenger Syndrome：ASD（3），VSD（8），PDA（3）  Other（2） | mild PAH：systolic PABP ≥30 and <50 mmHg on echocardiography32 or mean PABP ≥25 and <40 mmHg by catheterization  severe PAH： systolic PABP ≥50 mmHg on echocardiography or mean PABP ≥40 mmHg by catheterization | Week of delivery、Birth weight、Delivery mode、 (g)、SGA、General anesthesia、Delivered |
| Lai, Weisi et al. | 2021 | Moderate PAH= CHD（22），RHD（13），Hyperthyroidism（1），SLE（1）， PIH（2）  Severe PAH=CHD（37），RHD（10），Hyperthyroidism（0），SLE（0）， PIH（2） | Moderate PH：PSAP <50 mmHg  Severe PH ：PSAP ≥50 mmHg  . | Week of delivery，Method of delivery、General anesthesia 、Birth weight 、Induced abortion、Deterioration of NYHA class、Neonatal death、Neonates with asphyxia 、Class I-II、Class II-IV |
| LI Bin，et al. | 2013 | Rheumatic heart disease：Mild PAH：5；Moderate PAH and Sever PAH ：6  CHD：Mild PAH：29；Moderate PAH and Sever PAH ：63 | Mild PAH：30-49 mmHg  Moderate PH：50-79 mmHg  Severe PH ： ≥80 mmHg  . | Class I-II、Class II-IV、Term delivery、premature、Induced labour、Birth weight、Maternal Death、Neonatal asphyxia、Neonatal Death，Low birth weight |
| LI Ning，et al. | 2017 | CHD 46,Rheumatic heart disease 39 | Mid PAH：sPAP 30~49 mmHg  Moderate PAH：sPAP 50~79 mmHg  Severe PAH：sPAP ≥80 mmHg | Class I-II、Class II-IV、Mode of delivery、Induced labour |
| LIN Feng，et al. | 2009 | Mild PAH=CHD（24）、GDM（1）、Rheumatic heart disease（19）、HDCP（4）、Arrhythmia（3）、Hypertrophic cardiomyopathy（3）、SLE（1）、Other（2）  Moderate PAH= CHD（7）、GDM（2）、PIAH（2）、Rheumatic heart disease（10）、HDCP（2）、Arrhythmia（1）、Hypertrophic、SLE（1）  Severe PAH=CHD（6）、Rheumatic heart disease（3）、 | Mild PH：PASP ≥ 30 and ≤49 mmHg  Moderate PH：PASP ≥ 50 and ≤79 mmHg  Severe PH：PASP ≥80 mmHg | Neonatal death、NYHA class I-II、NYHA class III-IV、Maternal Death、Premature、Induced abortion、Mode of delivery、Term Delivery |
| LIN Jian-hua, et al. | 2006 | CHD：Slight PAH：19，Moderate PAH：16，Severe PAH：1  Rheumatic heart disease：Slight PAH：10，Moderate PAH：7，Severe PAH：4  Others：IPAH：4，SLE：1，arrhythmia：1 | slight PAH：PABP ≥30 and <50 mmHg  moderate PAH：PABP ≥50 and <80 mmHg  severe PAH： PABP ≥80 mmHg | Induced abortion、Premature、Neonatal asphyxia、NYHA class I-II、NYHA class III-IV、Term Delivery |
| LIN Li，et al. | 2013 | CHD：45；Rheumatic heart disease：30；IPAH,7,SLE:3 | Mild PAH：30-49 mmHg  Moderate PH：50-79 mmHg  Severe PH ： ≥80 mmHg  . | Week of delivery、Mode of delivery、Induced abortion；、Maternal Death、Neonatal asphyxia、Neonatal Death；Birth weight；premature |
| LIN Xiao,et al. | 2020 | IPAH 89,CHD 48,Rheumatic heart disease 13,Cardiomyopathy 1 | Mild PAH ：PASP 30-49mmHg  Moderate PAH：PASP 50-79mmHg  Severe PAH：PASP ≥80mmHg | Class I-II、Class II-IV、SGA、Neonatal asphyxia、Neonatal Death、Week of delivery、Mode of delivery |
| Luo, Jun et al. | 2020 | Mild PAH= CHD:ASD(22),VSD(11),PDA(1),Eisenmenger Syndrome：SLE(2)  Severe PAH=IPAH(1),CHD：ASD(5),VSD(10),PDA(4),ECD(4)  Eisenmenger Syndrome：ASD(1)、VSD（14），PDA（2）、SLE(2) | Mild PAH：50 mmHg ≥ SPAP >36 mmHg  Severe PAH：SPAP >50 mmHg | Week of delivery、Birth weight、Mode of delivery、SGA、Induced abortion、Premature、general anesthesia 、Class I-II、Class II-IV、Delivered、Fetal death、Neonatal asphyxia |
| Subbaiah, Murali et al. | 2013 | Mild PAH= CHD:ASD(8),VSD(6),PDA(2),  Severe PAH=IPAH(2),CHD：ASD(2),VSD(5),PDA(2),  Eisenmenger Syndrome：VSD（2），PDA（1） | Mild PAH：systolic PABP ≥30 and  ＜50 mmHg on echocardiography  Severe PAH: systolic  PABP ≥50 mmHg on echocardiography | Week of delivery、Birth weight、Mode of delivery、SGA、Deterioration in NYHA class、  Cardiac complications |
| WANG Wenjing，et al. | 2015 | CHD | Mid PAH：sPAP 30~49 mmHg  Moderate PAH：sPAP 50~79 mmHg  Severe PAH：sPAP ≥80 mmHg | Class I-II、Class II-IV、 Term of delivery，Premature，Neonatal asphyxia，Birth weight、Neonatal Death |
| WEI Ming-zhu，et al. | 2009 | Mild PAH=ASD（6）、VSD（5）、PDA（5）  Moderate PAH=ASD（2）、VSD（1）、TOF（1）、Rheumatic heart disease（8）  Severe PAH=ASD（1）、TOF（2）、Rheumatic heart disease（4） | Mild PH：PASP ≥ 30 and ≤49 mmHg  Moderate PH：PASP ≥ 50 and ≤79 mmHg  Severe PH：PASP ≥80mmHg | NYHA class I-II、NYHA class III-IV、Neonatal asphyxia、Premature、Induced abortion、Mode of delivery、Term Delivery |
| WU Yafeng，et al. | 2020 | CHD 49，Rheumatic heart disease 17，HDCP 13，Cardiomyopathy 8，Others 53. | Mild PAH ：PASP 30-49mmHg  Moderate PAH：PASP 50-79mmHg  Severe PAH：PASP ≥80mmHg | Class I-II、Class II-IV、Deterioration of NYHA class、Premature、Induced abortion、Neonatal asphyxia、Mode of delivery |
| YANG Dong，et al. | 2012 | CHD:20、Rheumatic heart disease：21、Cardiomyopathy：2、Systemic lupus erythematosus heart disease：1 | Mild PAH：30-49 mmHg  Moderate PH：50-79 mmHg  Severe PH ： ≥80 mmHg  . | Class I-II、Class II-IV、Term delivery、Premature、SGA、Mode of delivery、Induced labour、Maternal Death |
| YE Jun，et al. | 2010 | CHD:13、Rheumatic heart disease：3 | Mild PAH ：30–49 mmHg  Moderate PAH：50–79 mmHg  Severe PAH ：≥75 mmHg | Mode of delivery、Premature、Maternal Death、Birth weight、Class I-II、Class II-IV、Induced labour、Neonatal asphyxia、Neonatal Death |
| YUE Jun，et al. | 2011 | CHD:25、Rheumatic heart disease：11、arrhythmia：4、Perinatal cardiomyopathy：3 | Mild PAH：30-49 mmHg  Moderate PH：50-79 mmHg  Severe PH ： ≥80 mmHg  . | Class I-II、Class II-IV、Mode of delivery、Induced labour、Neonatal asphyxia、Neonatal Death、Premature、Maternal Death |
| ZHANG Chengju，et al. | 2018 | Mild PAH:CHD 13,HDCP 2，IPAH 1,Rheumatic heart disease 3  Moderate PAH:CHD 15,HDCP 2，Rheumatic heart disease 1，Connective tissue disease 1,  Severe PAH:CHD 21,IPAH 1,Rheumatic heart disease 3,pulmonary embolism 1，Connective tissue disease 1, | Mid PAH：RVSP 30~49mmHg  Moderate PAH：RVSP 50~79 mmHg  Severe PAH：RVSP ≥80 mmHg | Maternal Death、Week of delivery、NICU admission、Birth weight |
| ZHANG Xiao，et al. | 2021 | CHD:ASD 17,VSD 9,PDA 3,TOF 4,Combined defect 5 38,IPAH 6,Rheumatic heart disease 3,Heart valve disease 3，Perinatal cardiomyopathy 2 | Mild PAH ：PASP 30-49mmHg  Moderate PAH：PASP 50-79mmHg  Severe PAH：PASP ≥80mmHg | Class I-II、Class II-IV、Mode of delivery、Week of delivery、General anesthesia、Term of delivery、Premature、Induced abortion、ICU admission、Maternal Death、NICU admission、Neonatal death |
| ZHAO Chuchu,et al. | 2020 | CHD:ASD 46,VSD 25,TOF 5,PDA 10,ASD+VSD 1,ES 4,Endocardial cushion defect 4 | Mild PAH ：PASP 30-49mmHg  Moderate PAH：PASP 50-79mmHg  Severe PAH：PASP ≥80mmHg | Class I-II、Class II-IV、Term of delivery、Birth weight、Neonatal asphyxia、Neonatal Death、Premature、 |
| ZHOU Xiaorui，et al. | 2013 | Mild PAH：CHD:ASD 9,VSD 1,PDA 1,TOF 4,Ebstein 1,  Joint defect 1,others 0,Rheumatic heart disease,13  Moderate PAH:IPAH ；CHD:ASD,15,VSD 3,PDA 4,TOF 1,Ebstein 1,others 3,Rheumatic heart disease,6  Severe PAH:IPAH 4 ；CHD:ASD,21,VSD 37,PDA 9,Joint defect 6,others 1,Rheumatic heart disease,8 | Mild PAH：sPAP:30-49 mmHg  Moderate PAH：sPAP:50-74mmHg  Severe PAH ：sPAP: ≥75 mmHg | Premature、cardiac complications、Deterioration of NYHA class、Induced abortion |
| Zhou, Qian et al. | 2021 | Mid PAH=CHD（2）、GDM（2）、Hypertrophic cardiomyopathy（2）、Rheumatic heart disease（2）  Moderate PAH= Lupus nephritis（1）、CHD（1）、Bilateral renal leiomyoma（1）、GDM（1）、Heart failure （1）、hyperthyroidism（1）、Hypothyroidism（2）、Rheumatic heart disease（1）  Severe PAH=Lupus nephritis（5）、CHD（16）、Systemic scleroderma（1）、Heart failure （4）、Sjogren‘s syndrome（1）、Hypertrophic cardiomyopathy（1）、Rheumatic heart disease（1）、Systemic lupus erythematosus（1） | Mild PAH ：30–49 mmHg  Moderate PAH：50–79 mmHg  Severe PAH ：>79 mmHg | Gestational weeks at delivery ；Class I-II、Class II-IV |
| Zhu, Cai-Xia et al. | 2018 | NR | Mild PH：PASP ≥ 30 and ≤49 mmHg  Moderate PH：PASP ≥ 50 and ≤69 mmHg  Severe PH：PASP ≥70 mmHg | Gestational weeks at delivery、Mode of delivery、SGA,、Induced abortion,,  Premature、General anesthesia,、Postpartum hemorrhage、ICU admission、Class I-II、Class II-IV、Neonatal death,、Neonatal asphyxia、LBW、 NICU admission, |
| Budhram,S et al. | 2022 | NR | Mild PAH ：30–49 mmHg Moderate PAH：50–89 mmHg Severe PAH ：>89 mmHg | Maternal mortality, Premature, Method of delivery, Low birth weight infants |
| Su.J.Y et al | 2022 | The etiology of PAH included 47 cases (50.00%) of congenital heart disease, 18 cases (19.15%) of rheumatic heart disease, 7 cases (19.15%) of idiopathic PAH, 7 cases (7.45%) of hypertensive disease during pregnancy, 6 cases (7.45%) of the connective tissue disease systemic lupus erythematosus (SLE), 4 cases (6.38%) of hyperthyroidism heart disease, 3 cases (4.26%) of perinatal cardiomyopathy, 1 case (3.19%) of chronic pulmonary thromboembolism, and 1 case (1.06%) of anemic heart disease. | Mild PAH ：30–49 mmHg Moderate PAH：50–79 mmHg Severe PAH ：>80 mmHg | Maternal mortality, Premature, Cardiac complications, Birth weight, Neonatal asphyxia, Neonatal mortality, Method of delivery |
| Zhang,Lin et al | 2022 | NR | Mild PAH ：30–49 mmHg Moderate PAH：50–69 mmHg Severe PAH ：>70 mmHg | Maternal mortality, Cardiac complications, Neonatal preterm birth, Low birth weight infants, Neonatal asphyxia, Neonatal mortality, Mode of delivery, General anesthesia, NYHA III–IV |

Supplementary Table 2 NIH-QAT of the studies included in the comparison of mild and moderate to severe pulmonary hypertension

| **study** | **year** | **Q1** | **Q2** | **Q3** | **Q4** | **Q5** | **Q6** | **Q7** | **Q8** | **Q9** | **Q10** | **Q11** | **Q12** | **Q13** | **Q14** | **Quality Rating (Good, Fair, or Poor)** |
| --- | --- | --- | --- | --- | --- | --- | --- | --- | --- | --- | --- | --- | --- | --- | --- | --- |
| CHEN Lin, et al. | 2014 | Yes | Yes | Yes | Yes | No | Yes | NA | Yes | No | NR | Yes | NA | NA | NR | Fair |
| CHEN Yan, et al. | 2019 | Yes | Yes | Yes | Yes | No | Yes | NA | Yes | Yes | NR | Yes | NA | NA | Yes | Good |
| CHEN Yihong, et al. | 2012 | Yes | Yes | Yes | Yes | No | Yes | NA | Yes | Yes | NR | Yes | NA | NA | NR | Good |
| FAN Shu, et al. | 2020 | Yes | Yes | Yes | Yes | No | Yes | NA | Yes | No | NR | No | NA | NA | Yes | Fair |
| GOOG Yunxia, et al. | 2009 | Yes | Yes | CD | Yes | No | Yes | NA | Yes | No | NR | No | NA | NA | No | Fair |
| HAN Cha, et al. | 2013 | Yes | Yes | Yes | Yes | No | Yes | NA | Yes | Yes | NR | Yes | NA | NA | Yes | Good |
| HAO Jinhong, et al. | 2013 | Yes | Yes | Yes | Yes | No | Yes | NA | Yes | No | NR | Yes | NA | NA | NR | Fair |
| Katsuragi Shinji. et al. | 2012 | Yes | Yes | Yes | Yes | No | Yes | NA | Yes | No | NR | No | NA | NA | NR | Fair |
| Lai Weisi, et al. | 2021 | Yes | Yes | Yes | Yes | No | Yes | NA | No | No | NR | No | NA | NA | NR | Fair |
| LI Bin, et al. | 2013 | Yes | Yes | Yes | Yes | No | Yes | NA | Yes | No | NR | No | NA | NA | NR | Fair |
| LI Ning, et al. | 2017 | Yes | Yes | Yes | Yes | No | Yes | NA | Yes | No | NR | No | NA | NA | NR | Fair |
| LIN Feng, et al. | 2009 | Yes | Yes | CD | Yes | No | Yes | NA | Yes | No | NR | No | NA | NA | No | Fair |
| LIN Jian-hua, et al. | 2006 | Yes | Yes | CD | Yes | No | Yes | NA | Yes | No | NR | No | NA | NA | No | Fair |
| LIN Li, et al. | 2013 | Yes | Yes | Yes | Yes | No | Yes | NA | Yes | No | NR | No | NA | NA | NR | Fair |
| LIN Xiao, et al. | 2020 | Yes | Yes | Yes | Yes | No | Yes | NA | Yes | No | NR | No | NA | NA | Yes | Fair |
| Luo, Jun, et al. | 2020 | Yes | Yes | Yes | Yes | No | Yes | NA | No | No | NR | Y | NA | NA | Yes | Good |
| Subbaiah Murali, et al. | 2013 | Yes | Yes | Yes | Yes | No | Yes | NA | Yes | No | NR | No | NA | NA | NR | Fair |
| WANG Wenjing, et al. | 2015 | Yes | Yes | Yes | Yes | No | Yes | NA | Yes | No | NR | No | NA | NA | No | Fair |
| WEI Ming-zhu, et al. | 2009 | Yes | Yes | Yes | Yes | No | Yes | NA | Yes | No | NR | Yes | NA | NA | No | Fair |
| WU Yafeng, et al. | 2020 | Yes | Yes | Yes | Yes | No | Yes | NA | Yes | No | NR | Yes | NA | NA | Yes | Fair |
| YANG Dong, et al. | 2012 | Yes | Yes | Yes | Yes | No | Yes | NA | Yes | No | NR | Yes | NA | NA | NR | Fair |
| YE Jun, et al. | 2010 | Yes | Yes | Yes | Yes | No | Yes | NA | Yes | No | NR | Yes | NA | NA | NR | Fair |
| YUE Jun, et al. | 2011 | Yes | Yes | Yes | Yes | No | Yes | NA | Yes | No | NR | Yes | NA | NA | NR | Fair |
| ZHANG Chengju, et al. | 2018 | Yes | Yes | Yes | Yes | No | Yes | NA | Yes | Yes | NR | Yes | NA | NA | Yes | Good |
| ZHANG Xiao, et al. | 2021 | Yes | Yes | Yes | Yes | No | Yes | NA | Yes | No | NR | Yes | NA | NA | NR | Fair |
| ZHAO Chuchu, et al. | 2020 | Yes | Yes | Yes | Yes | No | Yes | NA | Yes | No | NR | Yes | NA | NA | NR | Fair |
| ZHOU Xiaorui, et al. | 2013 | Yes | Yes | Yes | Yes | No | Yes | NA | Yes | Yes | NR | Yes | NA | NA | Yes | Good |
| Zhou, Qian, et al. | 2021 | Yes | Yes | Yes | Yes | No | Yes | NA | Yes | Yes | NR | Yes | NA | NA | Yes | Good |
| Zhu, Cai-Xia, et al. | 2018 | Yes | Yes | Yes | Yes | No | Yes | NA | Yes | Yes | NR | Yes | NA | NA | Yes | Good |
| Budhram S, et al. | 2022 | Yes | Yes | Yes | Yes | No | Yes | NA | Yes | Yes | NR | Yes | NA | NA | Yes | Good |
| Su J Y, et al | 2022 | Yes | Yes | Yes | Yes | No | Yes | NA | Yes | Yes | NR | Yes | NA | NA | Yes | Good |
| Zhang Lin, et al | 2022 | Yes | Yes | Yes | Yes | No | Yes | NA | Yes | Yes | NR | Yes | NA | NA | Yes | Good |

Q1: Was the research question or objective in this paper clearly stated?

Q2: Was the study population clearly specified and defined?

Q3:Was the participation rate of eligible persons at least 50%?

Q4: Were all the subjects selected or recruited from the same or similar populations (including the same time period)? Were inclusion and exclusion criteria for being in the study prespecified and applied uniformly to all participants?

Q5: Was a sample size justification, power description, or variance and effect estimates provided?

Q6: For the analyses in this paper, were the exposure(s) of interest measured prior to the outcome(s) being measured?

Q7: Was the timeframe sufficient so that one could reasonably expect to see an association?

Q8: For exposures that can vary in amount or level, did the study examine different levels of the exposure as related to the outcome (e.g., categories of exposure, or exposure measured as continuous variable)?

Q9: Were the exposure measures (independent variables) clearly defined, valid, reliable, and implemented consistently across all study participants?

Q10: Was the exposure(s) assessed more than once over time?

Q11: Were the outcome measures (dependent variables) clearly defined, valid, reliable, and implemented consistently across all study participants?

Q12: Were the outcome assessors blinded to the exposure status of participants?

Q13: Was loss to follow-up after baseline 20% or less?

Q14: Were key potential confounding variables measured and adjusted statistically for their impact on the relationship between exposure(s) and outcome(s)?
